# CRISPR-Based Gene Editing in *Acinetobacter baumannii* to Combat Antimicrobial Resistance

**DOI:** 10.3390/ph16070920

**Published:** 2023-06-23

**Authors:** Muhammad Junaid, Krit Thirapanmethee, Piyatip Khuntayaporn, Mullika Traidej Chomnawang

**Affiliations:** 1 Department of Microbiology, Faculty of Pharmacy, Mahidol University, Bangkok 10400, Thailand; muhammad.jun@mahidol.ac.th (M.J.); krit.thi@mahidol.ac.th (K.T.); piyatip.khn@mahidol.ac.th (P.K.); 2 Antimicrobial Resistance Interdisciplinary Group (AmRIG), Faculty of Pharmacy, Mahidol University, Bangkok 10400, Thailand

**Keywords:** CRISPR-Cas, antimicrobial resistance (AMR), genome editing, *A. baumannii*

## Abstract

Antimicrobial resistance (AMR) poses a significant threat to the health, social, environment, and economic sectors on a global scale and requires serious attention to addressing this issue. *Acinetobacter baumannii* was given top priority among infectious bacteria because of its extensive resistance to nearly all antibiotic classes and treatment options. Carbapenem-resistant *A. baumannii* is classified as one of the critical-priority pathogens on the World Health Organization (WHO) priority list of antibiotic-resistant bacteria for effective drug development. Although available genetic manipulation approaches are successful in *A. baumannii* laboratory strains, they are limited when employed on newly acquired clinical strains since such strains have higher levels of AMR than those used to select them for genetic manipulation. Recently, the CRISPR-Cas (Clustered regularly interspaced short palindromic repeats/CRISPR-associated protein) system has emerged as one of the most effective, efficient, and precise methods of genome editing and offers target-specific gene editing of AMR genes in a specific bacterial strain. CRISPR-based genome editing has been successfully applied in various bacterial strains to combat AMR; however, this strategy has not yet been extensively explored in *A. baumannii*. This review provides detailed insight into the progress, current scenario, and future potential of CRISPR-Cas usage for AMR-related gene manipulation in *A. baumannii*.

## 1. Introduction

The behavioral change in bacterial pathogenicity leading to AMR is one of the major constraints hindering several public health policies globally. It has been estimated that there might be a global economic loss of USD 100 trillion by the year 2050 if this threatening issue of antibiotic resistance continues [1]. Although there are several reasons for antibiotic resistance, the major one is due to a bottleneck in innovative research with a focus on the exploitation of the diversity of antibiotics against Gram-negative bacteria, while the existing antibiotics are in clinical trials mostly under phase II or phase III. This lack of research is an alarming situation for research scientists along with economic loss. The lives of millions of people are projected to be vulnerable to death by 2050 due to the emerging situation of AMR [2]. Other serious health issues related to AMR include the outbreak of different infectious diseases, the risk of common infectious diseases such as immunosuppression, intubation, catheterization, and other such procedures related to antibiotics [3]. In view of the above situation, it is very important to revolutionize therapeutic strategies to prevent AMR bacterial infections [4,5]. It is critical to focus on the most commonly occurring pathogenic bacteria, which have acquired AMR for most antibiotics.

*Acinetobacter baumannii* is among the most widely distributed bacteria that can adapt to various environments. However, this pathogenic bacterium has been found to be resistant to all classes of antibiotics, and the WHO has emphasized the necessity of classification and research on this particular bacterium due to its high environmental adaptability [6]. Additionally, *A. baumannii is* highly resistant to hot and humid ultraviolet rays and chemical disinfectants and can survive on dry-surface objects for more than 25 days. *A. baumannii* can be traced to healthcare providers and to various dry surfaces, which can eventually be enablers of drug resistance [7].

Investigating different mechanisms for understanding drug resistance should be a high priority for research scientists. However, genetic manipulation is considered one of the robust approaches for studying such mechanisms in *A. baumannii* [8]. In addition, genetic manipulation of *A. baumannii* is applicable to laboratory strains ATCC17978 and ATCC19606, while it becomes less efficient for strains isolated from hospitals and patients because of their higher genetic diversity and increased AMR [9]. Various therapeutic tools have been developed recently, including peptides, bacteriophage therapies, antibodies, bacteriocins, and antibacterial or anti-virulence substances that are based on nucleic acids [10]. Along with the above genetic tools, CRISPR-Cas based gene editing offers an exciting opportunity for specific manipulation of the targeted genes responsible for antibiotic resistance in a specific bacterial strain. Several studies have been reported on the utilization of the CRISPR-Cas system for understanding the mechanism of AMR. This review will therefore focus on the previous studies in which CRISPR-Cas-based gene editing has played a vital role in overcoming the pressing issue of AMR and different bacterial strains including *A. baumannii.*

## 2. Clinical Significance of *A. baumannii*

*A. baumannii* is an opportunistic pathogen responsible for many diseases in humans, including pneumonia, skin infections, wound-borne infections, urinary tract infections, soft tissue infections, meningitis, and bacteremia. Among these infections, bacteremia and pneumonia are the most commonly reported infections, which have significant rates of morbidity and mortality [11]. One trait of some Acinetobacter strains that facilitates transmission through fomite contamination in hospitals is their ability to endure environmental desiccation for weeks. *A. baumannii* is responsible for nearly 80% of ICU-acquired pneumonia in many regions including Asia, the Middle East, and Latin America [12]. Globally, pneumonia is responsible for nearly 64% of mortalities in tropical regions and most frequently affects those with diabetes, lung disorders, and smoking or alcohol addiction [13]. Yet, it is still uncertain whether the main cause of infection is host factors or bacterial virulence factors [11]. In Europe and the U.S., ICU-acquired infections implicated by *A. baumannii* range from 2–10% and 2.5%, respectively, with a more than 50% resistance to various antibiotics [14]. The ranges for ICU-acquired infections by *A. baumannii* are 2.1% for skin/soft tissue infections, 1.6% for urinary tract infections, ~33% for wound infections, and 34.1% for bacteremia, with a 10–47% mortality rate [15]. The death toll due to *A. baumannii*-acquired bacteremia has been reported at 37–52%. Although the infestation rate for meningitis is not significantly high, it has a greater motility rate of nearly 70% [16]. It has been observed that in many Asian countries, 51% and 82% of nosocomial infections are caused by drug-resistant Acinetobacter isolates, respectively, where more than 80% infection rates were detected in India, Malaysia, and Thailand and ~59% in China [17]. The overall death rate by *A. baumannii* infections ranges from 30–43.4% in Thailand [18]. The following factors primarily contribute to acquiring *A. baumannii* infections: prolonged admission in hospitals and especially in intensive care units (ICUs), aging, multimorbidity, weak immunity, antibiotic usage history, injuries and burns, surgery, prematurity in newborns, use of contaminated equipment, mechanical ventilation, and permanent usage of catheters [13,19]. Moreover, natural or manmade disasters including wars, earthquakes, and tsunamis also contribute to *A. baumannii*-acquired infections, especially skin and soft tissue infections [20,21].

## 3. Antimicrobial Resistance Mechanisms in *A. baumannii*

Bacteria have evolved to develop several mechanisms to neutralize the effect of antibiotics due to the introduction of a large number of new antibiotics and their consequent consumption around the world [22]. Antibiotic resistance mechanisms in bacteria are broadly divided into three categories, namely intrinsic, acquired, and adaptive resistance [23]. The bacterial genome is the sole determinant of intrinsic resistance, which is typically acquired through drug inactivation, decreased membrane permeability for the medication, or enhanced efflux of the antibiotic, ultimately restricting access to the target [24]. *Moraxella catarrhalis*, *Salmonella typhimurium*, *Klebsiella pneumoniae*, *Pseudomonas aeruginosa* PAO1, and *Enterobacter cloacae* ATCC 13047 are examples of bacteria showing intrinsic resistance [25,26].

Genetic mutations or post-translational modifications help bacteria to acquire resistance. They enable bacteria to become resistant to a certain type of antibiotic to which they were previously susceptible. Genetic manipulation processes (transformation, conjugation, or transduction) can lead to the acquisition of certain genes that can develop resistant phenotypes in bacteria against antibiotics by mutation and selection [27]. Strains of *Escherichia coli*, *P. aeruginosa*, *A. baumannii, K. pneumoniae*, and *Vibrio cholerae* are common examples of bacteria with acquired resistance [28,29,30,31]. Bacteria can temporarily avoid antibiotic effects under adaptive resistance, as this resistance mechanism is produced under the effect of exogenous stimuli. The action of environmental stimuli results in transient genetic effects that hydrolyze or modify the antibiotic and ultimately result in the inactivation of its activity [28]. Inactivation or removal of these exogenous environmental signals reverses the resistance mechanism. Various environmental factors include changes in pH, organic compounds (carbon and polyamines), ineffective antibiotic doses, and anaerobiosis [32]. This type of resistance is commonly presented by *Salmonella enterica*, *S. enteritidis*, *E. coli*, and *P. aeruginosa* [33,34].

In *A. baumannii*, many strains present resistance to most of the existing antibiotics (Table 1). The genetic plasticity of *A. baumannii* enables it to produce higher genetic mutations and genetic rearrangements, along with the flexibility to integrate external elements into its genome through mobile genetic elements. In particular, insertion sequences are viewed as one of the fundamental mechanisms influencing how bacterial genomes and, ultimately, evolution are shaped. To resist different kinds of antibiotics, *A. baumannii* can use a variety of resistance mechanisms [35,36,37]. Yet, resistance to a specific antibiotic family can be produced due to the combination of various distinct resistance mechanisms. Moreover, *A. baumannii* has the ability to produce biofilms, which enables it to survive longer on medical equipment such as ventilators in ICUs. Although the connection between the development of biofilms and antibiotic resistance is not yet clear [38,39], the most common mechanisms in *A. baumannii* conferring resistance to multiple antibiotic families are plasmid conjugation, transposon acquisition, or integron mobilization. The functional insertion sequences are critical in enhancing AMR and gene plasticity in *A. baumannii* [40,41].

*A. baumannii* has been classified into multidrug-resistant (MDR), extensively drug- resistant (XDR), and pan drug-resistant (PDR) phenotypes depending on its ability to respond to various antibiotics [37]. The isolate is non-susceptible to at least one agent in at least three antimicrobial categories is referred to be MDR. *A. baumannii* isolates that pose non-susceptible to at least one agent in all but two or fewer antimicrobial categories are classified as XDR phenotypes, whereas the PDR phenotype is an isolate with non-susceptibility to all agents in all antimicrobial categories. Antimicrobial categories for *Acinetobacter* spp. include antipseudomonal carbapenems, penicillins with *β*-lactamase inhibitors, antipseudomonal penicillins with *β*-lactamase inhibitors, antipseudomonal fluoroquinolones, extended-spectrum cephalosporins, aminoglycosides, folate pathway inhibitors, polymyxins, and tetracyclines [42]. The use of carbapenems to treat MDR *A. baumannii* is no longer effective [43] and has been replaced with polymyxins to treat MDR *A. baumannii* infections; however, these drugs can have nephrotoxicity and neurotoxicity [44]. Aminoglycoside resistance genes, *β*-lactamases, and methyltransferases have contributed to the development of MDR phenotypes in *A. baumannii* [45]. Various mechanisms are adapted by *A. baumannii* to confer resistance against antibiotics. Efflux pumps or reduced permeability in bacteria hinder the access of antibiotics to the target site in the cell. In some cases, bacteria can inactivate the antibiotic using enzymes that can hydrolyze and modify the antibiotic’s structure. Moreover, genetic mutations or modifications can help bacteria modify the specific target sites for antibiotics and thus attain resistance [46,47,48]. These mechanisms enable *A. baumannii* to resist various antibiotic families, including *β*-lactams, aminoglycosides, tetracyclines, erythromycin, macrolides, polymyxins, chloramphenicol, fluoroquinolones, and trimethoprim [37,47,48].

*A. baumannii* strains present resistance to various antibiotics [49,50,51,52,53]. Among these, *β*-lactamases are the most common resistance mechanisms that are divided into four classes (A–D). Classes A, C, and D β-lactamases are the active-site serine *β*-lactamases, while class B has zinc or any other heavy metal-dependent or metallo-*β*-lactamases (MBLs) in the catalytic site. The *β*-lactams family includes penicillin, carbapenems, cephalosporins, cephamycins, and monobactams [54]. The strains having class A *β*-lactamase enzymes show resistance to all penicillin and cephalosporins but are less effective against cephamycin and carbapenems. This class is the most common source of *β*-lactam resistance. The genes associated with class A *β*-lactamase resistance in *A. baumannii* include *bla*PER-(1, 2, and 7), *bla*SHV-(5, 12, and33), *bla*GES-(11 and 14), *bla*TEM-(1 and 92), *bla*CARB-10, *bla*CTX-M-(2 and 15), *bla*SCO-1, and *bla*VEB-1 and also contain the *Klebsiella pneumoniae* carbapenemase (KPC) enzymes, including KPC-(2, 3, and 5*)* [35,37,55,56,57,58]. Class B or MBLs show resistance to almost all *β*-lactam antibiotics, including carbapenem, but cannot hydrolyze monobactams [59,60]. Globally, a variety of MBLs have been identified in *A. baumannii* [47,61]; however, detection of MBLs by conventional methods is not very effective, so there is a need to apply more molecular strategies, including next-generation sequencing (NGS), to detect MBLs [62,63,64].

All *A. baumannii* strains contain chromosomally encoded non-inducible cephalosporinases, which form class C *β*-lactamases. Class C is also recognized as Acinetobacter-derived cephalosporinase (ADC) induced by the *bla*ADC gene (formerly the *bla*AmpC gene) [65,66] and present resistance to penicillin, cefotenan, cephamycins, cefoxitin, and cephalosporins [48,59]. Class D *β*-lactamases can hydrolyze carbapenems and hence are known as carbapenem-hydrolyzing class D *β*-lactamase (CHLD) or oxacillinases (OXA). In *A. baumannii*, these lactamases can deactivate all *β*-lactams and provide resistance against carbapenem. The overexpression of OXA genes (chromosomal or plasmid encoded) enables *A. baumannii* to pose resistance against carbapenems [67]. Recent studies have shown the presence of various *bla*OXA enzymes (OXA-23, OXA-24, OXA-40, OXA-51, OXA-58, OXA-143, and OXA-235) in *A. baumannii* strains [68,69,70,71,72,73,74].

Enzymatic activity weakening the binding capacity of antibiotics, leading to changes in ribosomal target sites, efflux pumps, or permeability, provides resistance against aminoglycosides such as tobramycin, amikacin, and gentamicin in *A. baumannii* [75]. The aminoglycoside-resistant genes are specifically found in transposons, plasmids, chromosomal genomic islands, integrative conjugative elements, and chromosomes [76]. In *A. baumannii* strains, the role of efflux pumps involved in resistance against tetracyclines and tigecycline has been demonstrated by different studies [77,78,79,80,81]. Similarly, mutations in the *gyrA* gene are the main cause of resistance to fluroquinolones in *A. baumannii*; however, efflux pumps are also responsible for resistance to other groups of fluoroquinolones, including norfloxacin and ciprofloxacin [82]. *A. baumannii* has shown 50–73% resistance to fluoroquinolones, and in some regions, the resistance is up to 75–98% [83,84]. In addition, *A. baumannii* showed resistance to different macrolides, including erythromycin, oleandomycin, azithromycin, and clarithromycin [85]. It is resistant to polymyxins, especially exhibiting colistin resistance, which has significantly increased over time [86].

**Table 1:** Antimicrobial resistance mechanisms in *A. baumannii* against various antimicrobial categories.

Antimicrobial Categories	Resistance Mechanism	Class/Family/Activity	Enzymes/Genes/Proteins	References
*β-*lactams	*β*-lactamases	*Class A*	*Extended-Spectrum β-lactamases*	
			*bla*CARB-(4, 10)	[76,87]
			*bla*CTX-M-(2, 15, 43, 55, 115)	[88,89,90]
			*bla*PER-(1, 2, 3, 7)	[91,92,93]
			*bla*SHV-(5, 12, 33)	[57,94,95]
			*bla*VEB-(1, 3, 7)	[96,97,98]
			*bla*TEM-(1, 92, 116)	[99,100,101]
			*Narrow-Spectrum β-lactamases*	
			*bla*SCO-1	[102]
			*Carbapenem-Hydrolyzing β-lactamases*	
			*bla*GES-(1, 5, 11, 12, 14, 15)	[103,104,105]
			*bla*KPC-(2, 3, 5, 10)	[55,56,106]
		*Class B*	*bla*FIM-1	[107]
			*bla*GIM-1	[108]
			*bla*IMP-(1, 2, 4, 5, 6, 8, 10, 11, 14, 16, 19, 24)	[103,109,110]
			*bla*NDM-(1, 2, 3)	[111,112,113]
			*bla*SIM-1	[114]
			*bla*SPM-1	[115]
			*bla*VIM-(1, 2, 3, 4, 6, 11)	[116,117]
		*Class C*	*bla*AmpC-(69, 70, 71)	[118,119,120]
			*bla*ADC-(11, 25, 30, 56, 76, 152, 196, 222)	[121]
		*Class D* (Oxacillinases or OXA family)	*bla*OXA-(21, 37, 128) (*Narrow spectrum*)	[122]
			*bla*OXA-23 group, including *bla*OXA-(27, 49, 73, 102, 103, 105, 133, 134, 146, 165, 171,225, 239)	[104,123,124,125]
			*bla*OXA-24 group, including *bla*OXA-(25, 26, 27, 40, 72, 139, 160, 207, 40/24)	[90,126,127]
			*bla*OXA-48 group, including *bla*OXA-(48b, 162, 163, 181, 199, 204, 232, 247)	[128,129]
			*bla*OXA-51 group, including *bla*OXA-(64, 65, 66, 71, 75, 80, 82, 84, 86, 95, 98, 100, 104, 106, 113, 115)	[69,130,131]
			*bla*OXA-58 group, including *bla*OXA-(58, 96, 97, 164)	[9,41,132]
			*bla*OXA-143 group, including *bla*OXA-(143, 182, 231)	[133,134,135]
			*bla*OXA-235 group, including *bla*OXA-(235, 255)	[136,137]
Aminoglycosides	Overactive efflux pumps	Resistance nodulation division (RND)	*Ade*ABC, *Ade*FGH, *Ade*IJK, *Ade*R, *Ade*S	[47,69,138]
	Reduced membrane permeability		OmpA, Omp25, Omp33, OprB, OprC, OprD, OmpW, CarO	[139,140]
	Genetic mutations	Penicillin-binding protein (PBP)	PBP2, PBP3, PBP6b, *ftsI*	[47,141]
	Overactive efflux pumps	RND	*Ade*ABC, *Amv*A, *Ade*E, *Ade*R	[139,142,143]
	Genetic mutations	16sRNA methylase genes	*arm*A, rmt-(A, B, B1, C, D, E)	[139,141,143]
	Enzymatic inactivation	Aminoglycoside modifying enzymes (AME)	AAC, APH, ANT	[144,145,146,147]
Tetracyclines	Ribosomal protection	Dissociation of tetracycline from ribosome	Tet-M, Tet-O	[148]
	Overactive efflux pumps	RND and Tet pump	Tet-(A, B, C, D, G, H, M, X), *Ade*ABC, *Ade*IJK	[47,80,144]
Polymyxins	Genetic mutations	Lipid A, biotin	MCR-(1, 4, 4.3), *Pmr*CAB, Lps-(B, D), Lpx-(A, C, D), *pld*A, *Phe*S, *Znd*P	[47,149,150,151,152]
Macrolides	Overactive efflux pumps	Small multidrug resistance (SMR) pump	*Abe*S	[153]
Fluoroquinolones	Overactive efflux pumps	RND and multidrug and toxic compound extrusion	*Ade*ABC, AbeM	[154,155]
	Genetic mutations	DNA gyrase, quinolone resistance pentapeptide repeat protein	*Gyr*A, *Par*C, AAC, *Qnr-*(A, B, B19, S)	[83,84]

Retrieved and modified from [37,156].

## 4. Latest Strategies to Combat Antimicrobial Resistance in Bacteria

Recently, various strategies and genetic tools have been developed against AMR bacteria. These tools are utilized for the genetic screening and manipulation of bacterial genomes for AMR. The use of antibiotic markers (non-clinical and non-antibiotic) [152,157], antimicrobial peptides [158], transposon mutagenesis and screening (for high-throughput genetic screening) [159,160,161], anti-virulence compounds [162], suicide plasmids and linear DNA fragments (for gene deletion) [152,163,164], homologous recombination and complementation [165,166], phage therapy [166,167,168], nanoparticles [169,170,171], enzymes [54,172], drug repurposing [173,174,175], and vaccines [176,177,178] are common approaches to overcome AMR in bacteria.

RNA-based strategies such as RNA silencing and interference, antisense oligonucleotides, and steric-blocking oligonucleotides are also proven to be effective against AMR bacteria. Resistance genes can be eliminated by enzymatically targeting the mRNA with these oligonucleotides [179]. Translation can be ceased in bacteria by RNA silencing, which is a built-in process in many bacteria. The *cis*- and *trans*-regions bind to the complementary regulatory regions present on a single mRNA strand (also known as antisense sequences) to halt the translation of certain genes [180]. Furthermore, this technique utilizes antisense RNA sequences to monitor resistant genes and mutations by creating antisense oligonucleotides that are continually redesigned to ensure that resistance is not encountered [181,182]. It also helps to identify and knock-down the AMR genes and to detect the mode of action of novel antibiotics [181]. Two important drawbacks of the use of RNA as a therapeutic measure are the limited intracellular absorptions and chemistry-dependent toxicities [179].

Genome-editing tools can be utilized to combat AMR in bacteria. These tools use restriction enzymes to target and cut a specific DNA sequence; for example, restriction nucleases, zinc finger nucleases (ZFN), and transcription-activator-like effector nucleases (TALENS), are the initially developed genome-editing technologies that can be specifically engineered for target-specific DNA cleavage. These enzymes produce target-specific double-strand breaks (DSBs) in the genome and thus help to obtain knock-down, knock-in, and/or knock-out mutants. The cleavage domain present in these enzymes can bind to a customized DNA binding domain, which allows the DNA cleavage at the targeted binding site. Several studies have shown the utilization of both these enzymes in genome editing; yet, they are costly, laborious, time-consuming, and error-prone due to higher ratios of off-target mutations [183]. Moreover, TALENs are much larger in size than ZFNs, making them difficult to deliver and express in the host cells [184,185].

Many of the above-discussed genetic manipulation approaches are effective in genome editing, providing desirable gene deletions and mutations for antimicrobials; however, the limitations associated with these approaches necessitate the development and discoverer of novel alternative strategies with a more precise and target-oriented approach for genetic manipulation [186]. Recently, clustered regularly interspaced short palindromic repeats/CRISPR-associated protein (CRISPR-Cas) systems have become an effective tool to target drug-resistant bacteria and genes by targeting the specific genome sequences. CRISPR-Cas systems are being utilized to develop precise and effective antimicrobials for various infections; however, CRISPR-based approaches for AMR are still needed to be explored on a wider scale.

## 5. Clustered Regularly Interspaced Palindromic Repeats/CRISPR-Associated Protein (CRISPR-Cas) System

CRISPR-Cas constitutes an adaptive immune system as present in bacteria and archaea, which provides them with an effective shield against various viral and bacteriophagal attacks [187]. Discovered by Japanese scientists in 1987 and obtaining its name, CRIPSR, in 1990 by Francisco Mojica, the biological function and ability of these repetitive palindromic DNA sequences were long unknown [188]. Initially, in 2007, CRISPR was experimentally attributed as a crucial component of a prokaryote’s adaptive immune system in the fight against viruses [189]. Later, in 2012, Doudna and Charpentier discovered the role of CRISPR-Cas9 in DNA editing in a target-specific manner by using the appropriate template sequence. Moreover, they explained the processing mechanism of CRISPR-derived RNAs (crRNA) under the effect of transactivating CRISPR RNAs (tracrRNA) [190,191]. Since then, CRISPR-Cas has emerged as the most effective, precise, efficient, and powerful approach for editing the genome in all living cells and is used in a wide range of practical fields [190,192].

**Figure 1:**
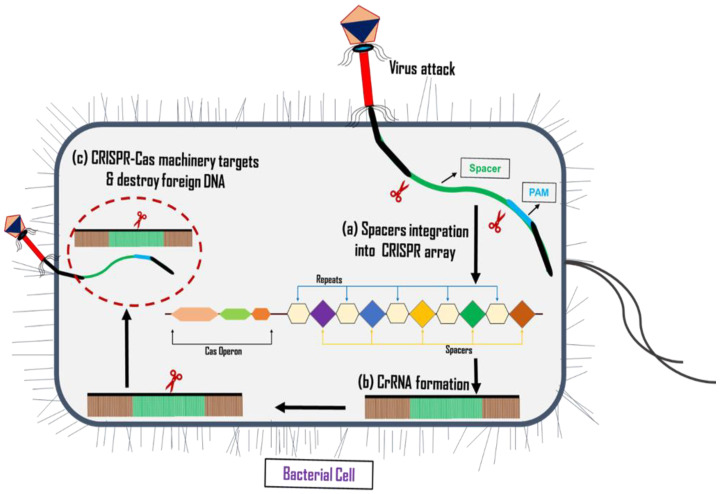
Schematic illustration of a three-stage adaptive immunity mechanism in bacteria using CRISPR-Cas machinery. (**a**) Adaptation/Acquisition: PAM sequence identification and protospacer integration into CRISPR-array by the *Cas* protein complex after viral invasion. (**b**) Transcription: production of CRISPR RNA (crRNA) molecules by transcription of CRISPR sequence. (**c**) Targeting/Interference: formation of crRNA + Cas nuclease complex, identification of the target invading sequence, and cleaving of foreign DNA to avoid infection.

Three fundamental steps of the CRSIPR-Cas system, which are adaptation (spacer acquisition), crRNA synthesis (expression), and target interference, play a vital role in bacterial defense against viral attacks (Figure 1). The *cas* gene encodes the Cas protein or nuclease protein, which is responsible for the cleavage and destruction of foreign viral DNA [193]. Its features, namely that it is an affordable, rapid, accurate, effective, and efficient method of gene editing, make CRISPR-Cas most researched genome-editing tool in recent years, where it has demonstrated the ability to get rid of bacterial infections [194,195], correct genetic flaws [196,197], and eradicate dangerous infectious viruses [198,199].

## 6. Classification of CRISPR-Cas System

Two main classes of the CRISPR-Cas system, Class 1 and Class 2, are distinguished by their signature genes and the arrangement of the CRISPR loci [200]. Both classes are further divided into 6 types (I–VI) and 33 subtypes. Class 1 comprises three types (I, III, and IV) and sixteen subtypes, whereas Class 2 includes three types (II, V, and VI) and seventeen subtypes.

In Class 1, when complexed with the crRNA, multi-subunit Cas effector proteins (Cas5, Cas7, Cas8, and SS) carry out the processing and interference mechanisms (where type I, III, and IV use Cas3, Cas10, and Csf1, respectively) to degrade the foreign DNA [201]. Nearly 60% of the bacterial population contains the Type I CRISPR-Cas system, which carries a multi-subunit CRISPR-associated complex for antiviral defense (Cascade) [202]. Seven subtypes (A to G) exist for Type I, among which the Type I-F CRISPR-Cas system is most prevalent among bacteria [200,203]. In the case of Class 2, a single enzymatic crRNA-binding protein is responsible for the identification and cleavage of the target sequence [201,204,205]. These multidomain crRNA-binding effector proteins include Cas9, Cas12a (Cpf1), and Cas13, which are responsible for the interference and processing mechanisms in type II, V, and VI, respectively [206]. CRISPR-Cas9 is isolated from the bacterium *Streptococcus pyogenes*, and in Type II CRISPR-Cas systems, it has received substantial attention from researchers for gene editing, as it is the easiest and most effective, versatile, and specific system [200,204].

## 7. Importance of CRISPR-Cas System

### 7.1. Role of CRISPR-Cas System against AMR Bacteria

Genome-editing technologies have been completely transformed by CRISPR-Cas due to its ability to be engineered to target almost any sequence of interest. As well as being repurposed for potential antimicrobials, CRISPR-Cas systems are also being used to reduce the level of undesirable genetic traits in bacteria [207,208]. Moreover, the ability of nucleic acid destruction by RNA enables the CRISPR-Cas system to develop next-generation antimicrobial mechanisms against infectious diseases, particularly those caused by AMR pathogens [209,210,211].

The CRISPR-Cas system based on the target gene locations can be employed as an antibacterial agent in two different ways, namely a pathogen-focused approach and a gene-focused approach [212]. In a pathogen-focused approach, particular bacterial chromosomal regions are targeted, where pathogen strain identification and bacterial cell death are achieved. On the other hand, targeting the plasmids that carry the AMR genes is part of the gene-focused approach. This method eliminates the plasmid and makes the bacterium susceptible to antibiotics [213,214]. The pathogen-focused approach is applied to cure distinct infections and can kill target-specific bacterial strains in a mixed culture, whereas the role of gene-focused approaches is still vague. These can be applied to the treatment of bacterial infections and can generally lessen the prevalence of the AMR gene in bacteria [206].

The CRISPR-Cas system, in contrast to conventional antimicrobials, operates in a target-specific manner, allowing the differentiation between friendly and harmful bacteria. Guide RNAs can be designed to target important virulence genes, antibiotic resistance, or pathogenicity [215,216]. According to the experimental designs and objectives, targeting effects can range from cell death to growth inhibition of the specific bacteria and at the genetic level can also result in gene deletions, transcriptional inhibition, and loss of antibiotic-resistant plasmids [217,218,219]. The CRISPR-Cas system utilizes the following three general strategies to address AMR:

1. It can be used in target-specific cleavage of infection-causing genes, deploying the desired bacteria while leaving the host’s microbiome unaffected [220,221]. For example, chromosomal genes for cell division and metabolism were removed from the mixed cell cultures of *E. coli* and *S. enterica* strains using the Type I CRISPR-Cas system [222];
2. It can be applied to cleave drug-resistant genes by killing the pathogenic bacteria but not affecting wild-types [222,223]. Bikard et al. applied the RNA-guided nuclease Cas9 against the virulence genes in *Staphylococcus aureus*, which resulted in the specific killing of virulent strain without affecting avirulent staphylococci [224];
3. It can be engineered to modify or silence resistance genes, causing bacterial mutations where the functionality of resistance genes is halted, while bacterial viability is maintained, known as the re-sensitization process [225,226]. The re-sensitization of *E. coli* strains using ESBL-encoding plasmids was carried out by Kim et al. [220]. They used plasmids encoding for Cas9 and crRNAs against conserved areas in the ESBL genes to transform strains of *E. coli* that produce ESBLs. The CRISPR-Cas9 system effectively reduced the resistance in the transformants by targeting specific cleavage of resistant plasmids. The realization of the broad utility of the CRISPR-Cas system in gene editing accelerated the need to search for Cas protein variants with enhanced functions, including higher activity, potential for therapeutic delivery, nucleic acid detection, etc. [227]. Among various Cas proteins, the most frequently used Cas proteins are Cas9, which results in a double-strand break by specifically cleaving the targeted sequence [214]; dCas9, a catalytically “dead or defective” Cas9 protein that contrasts with Cas9 by not showing double-strand nuclease activity, but instead staying attached to the targeted sequence and obstructing the RNA polymerase binding to that specific region, thud hindering the transcription initiation [228]; nSpCas9:rAPOBEC1, a Cas9 protein without nuclease activity attached to a deaminase, resulting in the conversion of cytidine bases into thymine and hence forming a stop codon [229]; and Cas13a, an RNA-specific endonuclease that, when recognized by particular DNA sequence, causes the cleavage of RNA fragments [5]. Cas14 is also attracting scientists’ attention as it is small, has single-stranded (ss) DNA-targeting activity, and does not require protospacer adjacent motif (PAM) sequences to bind, as compared to Cas9 and Cas12 proteins [227,230].

### 7.2. Recent Studies on the Application of CRISPR-Cas System in AMR Bacteria

Several academic studies have confirmed the application of the CRISPR-Cas system and its effectiveness in controlling and/or stopping of AMR in bacteria [231,232] (Table 2). The use of CRISPR-Cas13a to achieve cell death in *E. coli* strains against carbapenems and colistin-resistance genes was achieved through engineering and transfer of CRISPR-Cas13a via M13 phages [5]. Similarly, a trans-conjugative delivery system known as CRISPR Cas13a-based killing plasmids (CKPs) was applied to kill the endogenous AMR genes in *S. enterica* serotype Typhimurium, where colonies of *S. typhimurium* showed a substantial decrease through CRISPR-Cas13a [233]. More research can be performed on this strategy of employing Cas13a to target RNA transcripts to target the pathogenic bacterial strains depending on the presence of particular virulence genes.

AMR genes were successfully targeted in *E. coli* and *S. aureus* strains using a plasmid expressing a Cas9-driven RNA [212]. Clinical isolates of *S. aureus* were treated by manipulating the Cas9 and crRNA for the methicillin-resistance gene (*mecA*), which showed a significant (~50%) decrease in the disease as compared to the control [209,234,235]. Using the CRISPR-Cas system, a 20-time decrease in the number of viable cells of *E. coli* strain O157:H7 for the *eae* gene was observed, which is an essential gene for intestinal infections [219]. The application of the CRISPR-Cas9 system significantly reduced the *S. aureus* colonies on mouse skin in comparison to alternative treatment strategies [236]. A non-viral delivery was performed using a polymer, branched polyethyleneimine (bPEI), Cas9, and a single guide RNA (SgRNA) to combat the methicillin resistance gene, i.e., *mec*A, in *S. aureus*. The *S. aureus* strain treated with Cas9-bPEI did not grow in culture media, and growth was decreased by up to 32% compared to the control. These findings can lead to the development of novel CRISPR-based antimicrobial medications since the delivery of CRISPR via polymers can prevent risks of immunogenicity and off-target effects. It can also easily produce phenotypic alterations by editing and modifying the bacterial genome [237]. *E. coli* strains with resistance to colistin were developed by transforming the *mcr*-1 harboring *E. coli* strains with CRISPR-Cas9 plasmid, which not only eliminated the *mcr*-1 gene but also prohibited horizontal gene transfer after transformation with CRISPR-Cas9 plasmid [238]. In *E. faecalis*, the CRISPR-Cas9 system successfully targeted the erythromycin-resistance gene *erm*B and hampered growth, reducing the intestinal infections caused by this bacterium [239,240,241,242]. Many studies have shown the effectiveness of the CRISPR-Cas system for curing plasmids that present a resistant phenotype. This strategy can avoid horizontal gene transfer, target resistance genes to prevent AMR, and get rid of plasmid-carrying drug-resistant genes [234,243,244,245].

The CRISPR-Cas system can be used to explore the functions of various genes that can contribute to the increased antibiotic resistance in bacteria. The role of different genes in *K. pneumoniae* against tigecycline and colistin resistance was identified via the CRISPR-Cas9 system [246]. The study showed the knock-out mutants for the *tet*A gene presented a decrease in minimum inhibitory concentration (MIC) for tigecycline, whereas when the *mgr*B gene was inactivated, it resulted in the activation of the PhoPQ two-component system, ultimately increasing the MIC for colistin. *E. coli* strain SE15, responsible for biofilm formation in urinary catheters, resulted in reduced biofilm formation by targeting the quorum-sensing (QS) gene *lux*S through a CRISPR-Cas9 plasmid [247,248]. The CRISPR interference (CRISPRi) system, based on dCas9 (lacks the endonuclease activity compared to Cas9), is also used to target AMR genes. dCas9 forms a complex with sgRNA to bind at the targeted DNA sequence to inactivate the transcription and lead to gene silencing. Instead of a gene knock-out, the CRISPRi method is utilized to knock-down the desired gene, allowing for reversibility [249]. This strategy has been utilized for gene silencing and knock-down for the AMR genes in *E. coli* [250,251,252,253], *Enterococcus faecalis* [254,255], *Caulobacter crescentus* [256], *Campylobacter jejuni* [257,258], and other bacteria.

Newer strategies are also being explored, such as creating bacteriophages with DNA-encoding Cas9 and guide RNA and eliminating all phage sequences essential for phage replication. This approach will result in the cleavage and degradation of DNA in bacteriophages-infected bacteria through the CRISPR-Cas9 system; however, a continuous evolution of bacterial cells against foreign DNA might lead to the development of resistance to these approaches. Hence, bacterial mutations need to be researched, and phages must be designed to specifically target those mutations using the same strategy [259]. Moreover, CRISPR-Cas systems can be employed to either target and remove the AMR pathogen or to eliminate the bacteria themselves, which harbor the drug-resistant genes. As the evolution of bacterial resistance cannot be prevented, various CRISPR-Cas system-usage strategies can be evaluated further because the system is practical and easily reprogrammable [260].

**Table 2:** Application of CRISPR-Cas-based genome-editing strategies in various bacteria, including *A. baumannii*.

Genus	Bacterial Strains	Gram Staining	Targeted Gene/s	Resulted Modifications/Outcomes	References
Actinomyces		Gram +			
	Actinomycetes		*actIORF1* and *actVB*	Genome modification and gene inactivation and replacement	[261]
Acinetobacter		Gram –			
	*A. baumannii*		*bla*OXA-23, *bla*TEM-1D, and *bla*ADC-25	Genome editing and gene manipulation and deletions	[262]
	*A. baumannii* AB43		*Aba*I	Type I-F CRISPR-Cas system	[263]
	*A. baumannii*		*Adv*A and *fts*Z	CRISPRi, transposon mutagenesis, and gene editing	[264]
	*A. baumannii*		*glt*A and *β*-lactamase genes	Multiplex PCR and CRISPR-Cas12a	[265]
	*A. baumannii* AYE		*pyrF*	Genome editing, gene knock-out, and gene manipulation and deletions	[266]
Actinoplanes		Gram +			
	*Actinoplanes* sp. SE50/110		*Mel*C	Genome editing and gene deletions	[267]
Bacillus		Gram +			
	*B. subtilis*		*ku* and *ligD*	Genome alteration, DSB, and non-homologous end-joining (NHEJ) repair	[268]
	*B. subtilis*		*uppS*	CRISPRi and gene activity of essential genes	[269]
	*B. subtilis* ATCC 6051a		*amyE, aprE, nprE, spoIIAC*, and *srfC*	Genome editing and gene manipulation (up to 50%)	[270]
	*B. subtilis* 168		*trpc2*	Genome alteration, gene deletions, and point mutations	[271]
	*B. smithii*		*pyr*F	Genome modification, gene deletions, and silencing and insertions (90%, 100%, and 20%, respectively)	[272]
	*B. smithii* ET 138		*ldhL*	Genome editing, gene inactivation, and silencing with ThermoCas9 (active @ 55 °C)	[273]
	*B. licheniformis*		*yvmC*	Genome editing and gene knock-outs and integration	[274]
Brucella		Gram –			
	*B. melitensis*		*BE3*	Gene manipulation and 100% base replacement (C-T)	[275]
Campylobacter		Gram –			
	*C. jejuni* strains M1Cam and 81–176		*fla*A, *fla*b, astA, and *flg*R,	CRISPRi-based repression	[257]
	*C. jejuni* strains M1Cam and 81–176		*fla*A, *fla*b, and *flg*R,	CRISPRi-based gene repression	[258]
Caulobacter		Gram –			
	*C. crescentus*		*ctr*A and *gcr*A	CRISPRi and gene knock-downs	[256]
Clostridium		Gram +			
	*C. acetobutylicum* ATCC 824		*upp*	Genome editing and gene deletions, substitution, and insertions	[276]
	*C. acetobutylicum* DSM792		*hprK*	Genome editing and gene deletion and modifications	[277]
	*C. autoethanogenum*		*adh* and *2,3*-*bdh*	Genome editing and gene deletions	[278]
	*C. acetobutylicum* ATCC 824 and *C. beijerinckii* NCIMB 8052		*spoOA*	CRISPRi and genome deletion (*C. acetobutylicum* = 20 bp) (*C. beijerinckii* = 20–1149 bp)	[279]
	*C. beijerinckii*		*pta*	Genome modifications and single-nucleotide modification, deletion, and insertion	[280]
	*C. beijerinckii*		Amylase gene	CRISPRi and genetic manipulation (up to 97%)	[281]
	*C. botulinum*			Genome alteration and CRISPR-system presence analysis	[282]
	*C. cellulolyticum*		*afp*	Genome editing and gene deletion and integration	[283]
	*C. difficile*			Multiple genome-editing applications	[284]
	*C. difficile* JIR8094		*selD*	Genome editing and ~20–50% site-specific mutations	[285]
	*C. saccharoperbutylacetonicum* N1–4		*pta* and *buk*	Genomic modifications, gene deletions (~75%), and butanol production	[286]
	*C. pasteurianum*		cpa	Genome editing and gene deletion and insertion	[287]
Corynebacterium		Gram +			
	*C. glutamicum*		glgC, idsA, gltA, and *pyc*	CRISPRi	[288]
	*C. glutamicum*		*pyk* and *ldhA*	Base editor at different loci	[289]
	*C. glutamicum*		*ldhA*	Genome modification, gene deletion and insertion (~60%), and 80% gene modification	[290]
	*C. glutamicum*		*crtYf*	Genome editing and 86–100% successful deletions	[291]
	*C. glutamicum*		*clpX, mepA*, and porB	Genome editing, deletion, insertion, and point mutation	[292]
	*C. glutamicum* ATCC 13032		*argR, gabT,* and *gabP*	Genome editing and gene knock-out for gamma-aminobutyric acid (GABA) over-production	[293]
	*C. glutamicum*		*pgi*, *pck*, and *pyk*	CRISPRi (~98%)	[294]
Escherichia		Gram –			
	*E. coli*		*tal*B, *tkt*A, *xyl*A, and *xyl*B	Genetic manipulation, CRISPR, and enhanced xylose production	[295]
	*E. coli*		*sad*1, *sdh*A, *sdh*B, *suc*D, and *suc*C	CRISPRi	[296]
	*E. coli*		*aroA*	Gene replacements and insertions, point mutations, and deletions	[297]
	*E. coli*		*norVW*	Programmable DNA looping	[298]
	*E. coli*		*galK*, *lacZ,* and *pyrF*	Genome editing and simultaneous integration of 03 heterologous genes	[299]
	*E. coli*		*ackA, adhE, ldhA, maeA*, and *pta*	CRISPRi and increased malate production	[300]
	*E. coli*		*lac*Z	Genome editing, and gene replacement and insertions	[301]
	*E. coli*		*glt*A*, cat1, sucD, 4hbd, cat2, bld,* and *bdh*	CRISPRI, gene knock-out and knock-in, and 1,4-butanediol production	[302]
	*E. coli*		*gltA*	CRISPRi, genome modification, and *n*-butanol production	[303]
	*E. coli*		*arcAB* and *cpxR*	CRISPR-dCas9-based gene repression and multiple gene regulation	[304]
	*E. coli*		*soxR*	Genome engineering	[250]
	*E. coli*		*sul1*	CRISPRi	[251]
	*E. coli*		*Acr*A*, Acr*B, and *Tol*C	CRISPRi	[252]
	*E. coli*		*lux*S	CRISPRi	[253]
Enterobacter		Gram –			
	*E. hormaechei* 34978 and *E. xiangfangensis* 34399		*bla*KPC-3	Genome modifications and gene deletions	[243]
	*E. hormaechei* 4962		*bla*TEM-1	Genome editing and gene manipulation	[234]
Enterococcus		Gram +			
	*E. faecium* E745		*msr*C	Genome editing	[305]
	*E. faecalis* T11		pCF10	CRISPR based genome editing	[306]
	*E. faecalis* V583		pCF10	Genome manipulation	[307]
	*E. faecalis* CK135 and *E. faecalis* OG1SSp		*tet*M and *erm*B	Genome editing	[242]
	*E. faecalis*		*croR* and *ebp*A	CRISPRi and gene inactivation and silencing	[254]
Klebsiella		Gram –			
	*K. pneumoniae* Y4		*mgr*B	Genome modification and gene inactivation	[308]
	*K. pneumoniae* Y17		*tet*A and *ram*R	Genome modification and gene inactivation	[308]
	*K. pneumoniae* Kp97_58 and *K. pneumoniae* 13001		*bla*KPC-2	Genome modification and gene deletion	[243]
	*K. pneumoniae* 492110 and *K. pneumoniae* 5193		*bla*OXA-48 and *bla*OXA-48-like	Genome modification and gene deletion	[243]
	*K. pneumoniae* 3744 and 5573		*pyrF, fepB, ramA, fosA,* and *fepB*	Genetic manipulation using site-specific base editing	[229]
	*K. pneumoniae* KPCRE23		*bla*KPC-2, *bla*SHV, and *bla*CTX-M-65	Genetic manipulation using site-specific base editing	[229]
Lactobacilli		Gram +			
	*L. casei*		*LC2W_1326, LC2W_1628*, and *LC2W_2189*	Genome editing and gene deletions and integrations up to 25–60%	[309]
	*L. gassen*			CRISPR-Cas activity analysis in multiple strains	[310]
	*L. reuteri*			Efficient site-specific base alterations 90–100%	[311]
Mycobacterium		Gram +			
	*M. tuberculosis*		*pkn*B and *sig*H	CRISPRi and genetic modifications	[312]
	*M. tuberculosis*		*sigA*	CRISPRi and single/multiple targeted genetic modifications	[313]
	*M. tuberculosis*		*Sth*1	CRISPRi and gene inactivation	[314]
Pseudomonas		Gram –			
	*P. aeruginosa* PAO1 and *P. aeruginosa* PAK		*rhl*B, *rhl*R, and *prt*R		[315]
	*P. aeruginosa* PA154197		*mex*B, *mex*F, *mex*H, *mex*R, *mex*T, and *gyr*A		[138]
	*P. aeruginosa* PAO1 and *P. aeruginosa* PAK		*alg*R, *las*R, *nal*D, *rhl*B, *rhl*R, and *rsa*L		[225]
	*P. putida* KT2440		*ldhL*	CRISPRi-based genome editing	[273]
	*P. fluorescens Pf0-1, SBW25,* and *WH6*		*mNG, ftsZ*, and *mreB*	CRISPRi and gene silencing	[255]
	*P. aeruginosa*, *P. putida*, and *P. fluorescens*		*ftsZ*	CRISPRi-based genome editing	[316]
Staphylococcus		Gram +		Genome editing and gene inactivation	
	*S. aureus*		*agrA*, *cntA*, and *esaD*	Genome modification and base editing	[317]
	*S. aureus* RN4220		*erm*R and *mecA*	Genome editing and gene deletions	[318]
	*S. aureus*		*rfp*	Genome alteration and gene knock-out, insertion, knock-in, and single-base editing	[319]
	*S. aureus* CCARM, 3798, 3803, and 3877		*mecA*		[237]
	*S. aureus* 6538-GFP		*nuc*		[320]
	*S. aureus* AH1		*mec*	Type III-A CRISPR-Cas system for gene editing	[321]
	*S. aureus* ATCC 29213		*rpo*B	Genome modifications and gene deletions	[322]
	*S. aureus* USA300, USA300-∆mecA and RN4220		*mecA*	Genome editing and gene inactivation	[5]
	*S. aureus* USA300φ and *S. aureus* RNφ		*mecA*	Genome editing	[224]
	*S. aureus* ATCC 6538		*tar*H, *tar*G, and *tar*O	Genome alteration and gene knock-out	[228]
	*S. aureus* CTH96		*Nuc*	Genome editing and genetic manipulation and deletion	[323]
Streptomyces		Gram +			
	*Streptomyces*		Multiple genes	Multiplex gene disruption	[324]
	*S. coelicolor*			Genome editing and gene knocked-outs	[325]
	*S. lividans*, *S. albus*, *S. roseosporus*, *S. venezuelae*, and *S. viridochromogenes*		Biosynthetic gene clusters (BGCs)	Multiple genome editing and gene knock-in and gene insertion	[326]
	*S. coelicolor* M145		*actI-ORF2*	Genome editing and gene deletion (~900 bp)	[327]
	*S. avermitilis*		Ac*(3)Ⅳ*	Genomic disruption using Type I-E CRSIPR-Cas system	[328]
	*S. rimosus*		*zwf2* and *devB*	Genome editing, gene deletions, point mutations, and oxytetracycline production	[329]
	*S. lividans*, *S. viridochromogenes*, and *S. albus*		*sshg_05713*	Multiple genome editing and genome deletion (20 bp–30 kb)	[330]
	S. *coelicolor* A3(2)		*actIORF1* (SCO5087) and *actVB* (SCO5092)	CRISPRi and gene deletion	[261]
	*S. coelicolor*		*actII-orf4*, *redD*, and *glnR*	Genome editing and single- and multiple-gene deletions	[331]
Synechococcus		Gram –			
	*S. elongatus* UTEX 2973		*nbla*	Genome editing and gene deletion	[332]

Retrieved and modified from [333,334].

### 7.3. CRISPR-Cas System in A. baumannii

The existence of two endogenous CRISPR-Cas systems in the genomes of various *Acinetobacter* species has been verified by the analysis of the CRISPRCas database (*CRISPRCasdb*) [335,336,337]. Nearly 2500 genomes of *A. baumannii* subjected to a pangenome study also confirmed the presence of two CRISPR-Cas systems in *Acinetobacter* spp. [338]. The first system is present in the genome of clinical isolates of *A. baumannii* AYE, AB037, 4190, and AB0057, whereas the second system was discovered in *A. baumannii* type strain ATCC^®^19606^TM^ and *A. baylyi* ADP1 [339,340,341]. The CRISPR system is found in a variety of prokaryotes [337]. However, only 36% of the bacteria comprise both CRISPR arrays and Cas genes. The *CRISPRCasdb* analysis revealed the presence of CRISPR arrays and Cas genes in nearly 20% of organisms in the *Acinetobacter* genus and 18% of isolates of *A. baumannii* spp. [337,342]. The CRISPR system has been identified in different *A. baumannii* strains by analyzing the large volume of sequencing data and by application of bioinformatical tools [340]. The trailer and spacer regions of the CRISPR system are generally conserved among various bacterial isolates. This helps in the grouping of isolates and the identification of common ancestors based on the presence of sequence arrays [343]. Several studies have shown that the CRISPR-Cas system not only provides immunity in *A. baumannii* but also regulates various virulence gene expressions, controls group behaviors, provides DNA repair, and dictates genome evolution [342].

The Type I CRISPR-Cas system is the most common in nature, comprising a multi-subunit effector complex [200,344]. This effector complex includes nine subtypes known as A, B, C, G, D, E, F1, F2, and F3 [200]. Type I-F CRISPR-Cas systems are the most common in *A. baumannii* [337], but the Type IV variant with genes csf3, csf4 (also named as *din*G), and cas6e, along with CRISPR arrays at both ends, is also present in some *Acinetobacter* spp. [345]. Recently, several studies confirmed the presence of the Type I-F CRISPR-Cas system in various *A. baumannii* isolates from throughout the world [346,347,348,349,350]. Based on the presence or absence of the 14 common genes, the *Acinetobacter* genome can be divided into two groups. The first group comprises fewer common CRISPR genes and hence shows rarity in the presence of plasmids [338]. The existence of Type 1 and Type IV CRISPR-Cas systems in *A. baumannii* was also confirmed. In silico analysis of 4977 *A. baumannii* genomes from the NCBI Refseq database revealed nearly 14% of *A. baumannii* clinical isolates carried CRISPR-Cas systems [351,352,353]. Further classification of *A. baumannii* genomes presenting CRISPR-Cas systems showed that Type I-F1 CRISPR-Cas system was most abundant (~67%), followed by Type I-F2 (~28%), while both Type I-F1 and Type I-F2 were present in ~4% genomes. Various studies have reported the coexistence of different types of CRISPR-Cas systems in other bacteria [345,354,355,356]; however, the co-localization of Type I-F (I-F1 + I-F2) in *A. baumannii* was reported by Yadav and Singh [353].

The CRISPR-Cas system, specifically Type 1, is successfully employed for genome editing in various bacterial strains. There is still great potential to explore and implement it for genetic manipulation in *A. baumannii* by exploring the association of CRISPR-Cas systems with bacterial virulence and pathogenesis mechanisms [357,358]. Tyumentseva et al. confirmed the existence of CRISPR arrays and Cas genes related to Type I-F2 in clinical isolates of *A. baumannii* [342]. They also found a correlation between the AMR genotype/phenotype of *A. baumannii* with its type of CRISPR-Cas system. It was observed that a higher number of AMR genes was present in the isolates where both CRISPR arrays and active Cas genes were missing as compared to the isolates with only CRISPR arrays or both CRISPR arrays and Cas genes. This helps bacteria fight against phage infections and protect against the spreading of AMR genes in *A. baumannii*. Virulence factors were also found to be dependent on CRISPR-Cas systems in *A. baumannii*. Additionally, regarding the difference between Type I-F1 and Type I-F2 CRISPR-Cas systems among the isolates of *A. baumannii*, CRISPR arrays were lower in isolates with Type I-F1 CRISPR-Cas system as compared to Type I-F2 isolates, which have a stronger immune system. The presence of more AMR genes in Type I-F1 *A. baumannii* isolates supports easier adaptation to different environmental conditions, whereas Type I-F2 isolates may utilize the CRISPR-Cas system to control the distribution of AMR genes. Similarly, the Type I-F1 CRISPR-Cas system affects the acquisition of AMR plasmids in wild-type antimicrobial-susceptible *E. coli* isolates and sustains the susceptible profile of these *E. coli* isolates [359]. Other studies also confirmed the impact of the CRISPR-Cas system on the accretion of virulence and AMR-related genes, where it can prevent the accumulation of resistance genes but does not affect the mutations in cells to attain AMR. However, the *A. baumannii* genomes having CRISPR-Cas systems did not show any correlations with any specific antibiotic classes and with virulence genes [352], indicating no effect of this system on the acquisition of resistance and virulence genes in *A. baumannii*. The genomes possessing only co-localizing Type I-F1 + F2 CRISPR-Cas systems showed negative correlation for factors including biofilm-associated proteins (bap, *bau*A) and quorum-sensing (QS) genes (*aba*I and *aba*R) [353]. Similar results were also presented by other research groups [338,342,360]. These association analyses suggest that without affecting the phage-based memory, spacers can target plasmids through an unknown mechanism for the acquisition of virulence and resistance factors in the genomes of *A. baumannii*.

### 7.4. Recent Studies on the Application of the CRISPR-Cas System in A. baumannii

To apply an effective exogenous CRISPR-Cas system to *A. baumannii*, many researchers have generated and tested various models. An exogenous recombination system involving two plasmids carrying Cas9 from *S. pyogenes* and sgRNA was developed, where each plasmid could replicate in both *E. coli* and *A. baumannii*. The impact of the genes (*bla*OXA-23, *bla*ADC-25, and *bla*TEM-1D) was reported on imipenem and sulbactam resistance in *A. baumannii*. Additionally, the researchers constructed single-, double-, and triple-gene mutants to explain the role of each gene in accomplishing AMR [262,361]. The application of CRISPR-Cas-mediated genome modifications in *A. baumannii* may be tricky, as the procurement of several resistance genes is related to mobile genetic elements such as plasmids and transposons. Hence, the loss of the plasmid carrying the CRISPR components and unwanted genetic combinations might be obtained [229]. In this regard, an alternate strategy to perform gene editing is cytidine-base editing (C to T replacement), which does not require a DSB and a donor template. In this process, single-base replacement (A/C to T) in CAA or CAG can produce stop codons (TAA or TAG). The successful application of cytidine-base editing was achieved in *K. pneumoniae* and *A. baumannii* ATCC^®^17978^TM^ by constructing the plasmid vector pBECAb-apr with a sgRNA and a fusion protein expression [229]. Moreover, a two-plasmid-based CRISPR-Cas system to perform gene editing in *K. pneumoniae* [262] was tested in *A. baumannii*.

The relationship between drug resistance and the CRISPR-Cas system was analyzed in *A. baumannii* strain AB43 using the whole-genome sequencing (WGS) technique [362]. The authors identified the presence of the Type I-Fb CRISPR-Cas system in the strain AB43 and found that the *cas* gene in the studied strain has a higher similarity index with the similar subtype *cas* genes. The role of the CRISPR-Cas system in the AB43 strain against invasive bacteriophage and plasmids was confirmed, as 28 out of 105 CRISPR spacers in the genome of this strain showed similarity with the genes present in the bacteriophage genome and with the plasmid database. However, no matches for CRISPR spacers were found for AMR genes for *A. baumannii* strain AB43. The drug resistance mechanism in *A. baumannii* strain AB43 via the CRISPR-Cas system is still unclear, as the endogenous CRISPR-Cas system might be responsible for inhibition of drug resistance gene expression, which requires further research [342,362]. Another study using WGS identified the CRISPR-Cas system Subtype I-F in *A. baumannii* strain ATCC BAA1605, with a high number of spacers present in the CRISPR loci [363]. The *aba*I gene responsible for biofilm formation through the quorum sensing in *A. baumannii* [364] was targeted to develop gene knockouts by designing sgRNAs using various bioinformatical tools [365]. To target the essential genes in *A. baumannii* ATCC 17978, a CRISPRi-system was applied to develop gene knock-down mutants using the anhydrotetracycline (*a*Tc)-inducible dcas9 gene and a constitutive sgRNA for *adc β*-lactamases [264]. A significant (30-fold) decrease in *β*-lactamase synthesis was observed after the induction of aTc by dcas9, indicating successful silencing of the *adc* gene. The researchers also developed gene knockdowns for essential genes for cell replication, i.e., *fts*Z and *adv*A, using the CRISPRi approach and observed substantial decrease in cell growth as compared to the control. The CRISPRi approach was also used to characterize the transcriptional factor RS03245 encoding AraC in *A. baumannii*, which is important for bacterial growth. CRISPR-Cas12a array along with multiplex polymerase chain reaction (PCR) was implemented to detect MDR *A. baumannii*, enabling simultaneous amplification of essential genes and *β*-lactamase genes. This study showed the accuracy and specificity of the CRISPR-Cas12a system to detect important drug-resistance genes in *A*. *baumannii* [265].

An effective, convenient, and quick gene-manipulation system comprising *pyr*F-based suicide plasmids and *pyr*F-deleted uracil-auxotrophic hosts was developed and tested to successfully to delete the sequences of *cas* genes (*cas1*, *cas3*, and cascade) and the CRISPR sequence (except the leader and a single repeat structure) in the I-F CRISPR-Cas system in *A. baumannii* AYE∆F. This system is more efficient for developing knockouts in model strains than the clinical strains of *A. baumannii* due to lower transformation rates and biofilm formation [266]. The role of *Oxy*R as an oxidative-stress-resistance regulator was also discovered using the CRISPR-Cas9 system. The CRISPR-Cas9-based genome-editing strategy (pCasAb-pSGAb) involved Cas9 and *Rec*Ab from *A. baumannii*. The amount and the length of the repair template were also optimized, which resulted in significant improvement in editing efficiency. The genome-editing efficiency of strains ATCC^®^17978^TM^ and ATCC^®^19606T in one clinical isolate, *A. baumannii* ABH2, was also tested. The researchers used multiple strains because of the anticipated variation in the effectiveness of CRISPR-Cas-based genome editing due to differences in genomic background. An exogenous CRISPR-Cas system was used to introduce point mutations into a clinical isolate of *A. baumannii* ABH2 to study the role of H_2_O_2_-sensing amino acid residues present in *Oxy*R. As anticipated, the mutant strains did not exhibit any deficiencies with regard to H_2_O_2_ sensitivity. However, a known residue (C_2_0_2_) and three new residues (E130, S133, and S226) were identified to be important for *Oxy*R function [262]. In another study, the use of *Rec*Ab from the *A. baumannii* IS-123 strain produced more colonies by deleting *Oxy*R via CRISPR-Cas [366]. The CRISPR-based genome-editing system (pCasAb-pSGAb) in comparison to the gene-editing strategy (*pyr*F/5-FOA) developed by [266] showed higher deletion efficiency. However, the utilization of a single plasmid and its easier removal from cells in the *pyr*F/5-FOA system make it a more convenient method.

## 8. Conclusions

CRISPR-Cas-based gene editing has provided successful results for gene manipulation in various bacteria to combat AMR; affect various important physiological processes that cause infections and reduce virulence, pathogenicity, and biofilm formation; and can lead to bacterial death. However, the application of CRISPR-Cas in *A. baumannii* is quite recent and not fully explored. This revolutionary technique can be used to improve the genetic makeup of *A. baumannii* to combat AMR by targeting and disrupting specific genes associated with AMR, such as enzymes producing genes that protect the bacteria from antibiotics and biofilm-formation genes that provide antibiotic resistance. Moreover, the CRISPR can be utilized to modify the existing genes or introduce new genes into the genome of *A. baumannii* to reduce the bacteria’s ability to resist antibiotics or to increase its sensitivity to antibiotics. For example, a specific gene associated with a receptor that regulates the activity of an antibiotic could be introduced into the bacteria to enhance its susceptibility to that specific antibiotic. Similarly, the introduction of genes into the bacterial genome that produce specific molecules that interfere with AMR mechanisms and bacterial growth can be achieved through CRISPR-based genetic manipulation. Overall, CRISPR-based gene editing could be effectively used to combat AMR in *A. baumannii*, and the method still requires more research in this regard in the future.

## Data Availability

Not applicable.
